# Discussing sexuality with Parkinson’s disease patients: a multinational survey among neurologists

**DOI:** 10.1007/s00702-019-02053-5

**Published:** 2019-08-08

**Authors:** F. B. B. de Rooy, C. Buhmann, B. Schönwald, P. Martinez-Martin, C. Rodriguez-Blazquez, H. Putter, H. W. Elzevier, A. A. van der Plas

**Affiliations:** 1 Department of Urology, Leiden University Medical Center, Albinusdreef 2, 2300 RC Leiden, The Netherlands; 2 Department of Neurology, University Medical Center Hamburg-Eppendorf, Martinistraβe 52, 20246 Hamburg, Germany; 3 National Center of Epidemiology and CIBERNED, Institute of Health Carlos III, Madrid, Spain; 4 Department of Medical Statistics, Leiden University Medical Center, Albinusdreef 2, 2300 RC Leiden, The Netherlands; 5 Department of Medical Decision Making, Leiden University Medical Center, Albinusdreef 2, 2300 RC Leiden, The Netherlands; 6 Department of Neurology, Alrijne Hospital, Simon Smitweg 1, 2353 GA Leiderdorp, The Netherlands

**Keywords:** Parkinson’s disease, Practice patterns, Sexual dysfunction, Quality of life, Multinational

## Abstract

Sexual dysfunction is a major non-motor feature of Parkinson’s disease (PD) that may affect the quality of life of many patients. In a Dutch survey, we demonstrated that neurologists often fail to discuss sexuality with their patients. Our objective was to determine to which extent neurologists in Spain and Germany address sexuality with their patients and whether cross-cultural differences exist. A 30-item questionnaire was sent out to 1650 German and 460 Spanish neurologists. The questionnaire addressed attitudes, knowledge, barriers, and feelings of responsibility regarding sexuality in PD. 160 German and 32 Spanish respondents completed and returned the questionnaire. The majority of German and Spanish participants discuss sexual dysfunction ‘regularly’ with male patients (61.7% and 78.9%, respectively), but ‘seldom’ with female patients (68.8% and 78.1%, respectively). Important barriers for German and Spanish respondents to discuss sexual dysfunction were patients not expressing sexual complaints spontaneously (52.9% and 75.0%, respectively) and insufficient consultation time (32.2% and 71.9%, respectively). Sexual dysfunction in PD was considered important by 68.3% of German and 96.9% of Spanish participants. German and Spanish neurologists do not routinely discuss sexual dysfunction with their patients, although many of them consider it important to address this topic. It is unclear why this lack of discussing sexual dysfunction is especially found for female patients and whether cultural aspects are involved. We recommend a self-assessment tool for patients to track their symptoms prior to consultation visits and advocate local guidelines that formulate who is responsible for discussing sexual dysfunction.

**Electronic supplementary material:**

The online version of this article (10.1007/s00702-019-02053-5) contains supplementary material, which is available to authorized users.

## Introduction

Parkinson’s disease (PD) is a heterogeneous neurodegenerative disorder that encompasses both motor and non-motor symptoms (NMS) (Kalia and Lang 2015; Chaudhuri et al. 2006a, b). The spectrum of PD related NMS is broad and includes sexual dysfunction (SD), which comprises a variety of different presentations as well (Bronner 2011). The impact of NMS in PD is increasingly acknowledged, as symptoms may occur long before the first motor symptoms arise and usually remain throughout the course of the disease (Chaudhuri et al. 2006a, b; Schrag et al. 2015). This has also been demonstrated for erectile dysfunction in PD patients (Schrag et al. 2015; Noyce et al. 2016). The prevalence of NMS in PD patients compared to age-matched controls is high (Chaudhuri et al. 2006a, b), with reports of up to 98.6% of PD patients complaining of one or more NMS across all stages of the disease (Barone et al. 2009). A high prevalence has also been found for altered interest in sex and problems during sexual intercourse (57% and 66% of PD patients, respectively) (Santos-Garcia and Fuente-Fernandez 2013). Finally, the negative impact of NMS, including SD, on the quality of life of PD patients (Duncan et al. 2014; Laumann et al. 1999; Buhmann et al. 2017), further emphasizes the importance of screening for these symptoms during daily clinical practice. In a survey among Dutch neurologists specializing in PD, we found that the majority of participants considered screening for SD in PD patients’ essential. However, most of them also reported that they often omit to discuss sexuality with their PD patients (van Hees et al. 2017). Barriers that were particularly mentioned to discuss this topic included high age of patients, insufficient consultation time, and patients not raising the topic themselves. Although PD affects people across all racial groups, several studies have reported ethnic variations regarding the presentation of NMS, SD in particular (Sauerbier et al. 2017; Yu et al. 2017). The question arises whether the same accounts for neurologists from different countries with respect to their daily clinical practice, knowledge, and attitudes regarding discussing sexual health in PD patients. As such, we performed a survey among neurologists, who treat patients with PD in Germany and Spain, added the response of their Dutch colleagues (van Hees et al. 2017), and finally compared the results found in the three different countries. By determining existing barriers, perspectives on responsibility for discussing SD, current level of knowledge and potential need for training, possible recommendations can be made to improve sexual health care in PD patients.

## Methods

### Data collection

A cross-sectional study was performed among German and Spanish neurologists specializing in treating PD patients. In German, 1650 specialists were considered suitable to participate and received an invitation to join our study. These specialists, who in some cases were both neurologist and psychiatrist, were selected with the help of the German Parkinson Patient Organization, which named PD specialists for each of the 16 federal states. In Spain, the 460 members of the Movement Disorders Study Group of the Spanish Neurological Society (all neurologists) were contacted.

Questionnaires along with information and invitation letters were sent by regular post or email, dependent on the contact information that was available. Four weeks later, a reminder was sent to non-responders. Another 6 weeks later, a final reminder was sent to physicians who still had not responded. Both in Germany and Spain, no formal ethical approval was required, as the survey was addressed to professionals, not to patients, lacks personal data and research characteristics were non-invasive, non-harmful, and non-sensitive.

### Survey design

The content of the questionnaire was similar to the survey used in our previous Dutch study (van Hees et al. 2017) and translated into German and Spanish. This survey was based on questionnaires used in the previous studies concerning the discussion of sexuality in other medical departments (Korse et al. 2016; Krouwel et al. 2015; Nicolai et al. 2013; van Ek et al. 2015) and designed with the support of an academic neurologist specializing in PD and the Dutch PD Patient Association. Thirty multiple-choice questions were formulated with the possibility of adding free text in some questions (see Online Resource 1). Our interest primarily focused on the extent to which sexuality is discussed and possible barriers that exist for neurologists towards discussing SD. The questionnaire also covered topics such as the participant’s perspective on who is responsible for discussing SD, the use of the Parkinson Monitor and NMS scales, the organization of sexual health care, possibilities for referral, a self-assessment of knowledge on SD, and the need for implementing this topic into training schedules. The questionnaire also contained questions concerning demographic data. Respondents were offered an option to reject participation and were asked for reasons why they refused to participate.

### Statistical analyses

Quantitative data were analysed using SPSS Statistics 23 (SPSS Inc., Chicago, IL, USA). Demographic variables, as well as responses to the questionnaire, were described using descriptive statistics. Pearson’s Chi-square test or Fisher’s exact test were used to calculate associations between categorical data. As the Shapiro–Wilks test showed a normal distribution, numerical data were described as mean (standard deviation). One-way ANOVA was used to assess associations between numerical data of three groups. Some response options were grouped together into smaller categories for analyses. For example, the answers “Never/almost never” and “In less than half of the cases” were combined, as well as “Totally disagree” and “Disagree”. Two-sided *p* values of < 0.05 were considered statistically significant.

## Results

### Survey responses

In Germany, 216 out of 1650 neurologists (13%) returned the survey, of which 160 neurologists completed the entire survey or answered at least 88% of the questions, and as such, were considered suitable for further analysis. In Spain, 32 out of 460 neurologists (7%) who were asked to participate eventually replied. All 32 returned questionnaires were considered suitable to analyse, as they were filled in completely or almost completely.

### Demographics

Demographic data are presented in Table 1. Similar to the Dutch study, the majority of respondents in Germany were male (75.6%), whereas in Spain, most of the returned surveys were filled in by female neurologists (65.6%). The mean age of the participants was 54.3 (8.2) and 50.1 (9.3) years in Germany and Spain, respectively. In Germany, 69.7% of the respondents worked in a private practice, while 62.5% of the Spanish participants treated their patients in a university hospital. Gender and age of non-respondents were unknown for both countries.

**Table 1:** Demographic characteristics of participants (*n* = 280)

	Germany	Spain	The Netherlands
	*n *=160	*n *= 32	*n *= 88
Gender (%)			
Male	75.6	34.4	63.6
Female	24.4	65.6	36.4
Age in years, mean (standard deviation)			
Total	54.3 (8.2)	50.1 (9.3)	46.6 (8.5)
Male participants	55.6 (7.5)	52.7 (11.9)	48.7 (8.9)
Female participants	50.1 (8.9)	48.7 (7.7)	43.0 (6.5)
	*n *=159	*n *=32	*n *=88
Years of practice in neurology (*n* = 279), (%)			
< 1	0.6	3.1	0
1–2	0.6	0	10.2
3–5	1.3	0	14.8
6–10	3.1	9.4	30.7
11–15	8.8	18.8	14.8
> 15	85.5	68.8	29.5
	*n *=152	*n *=32	*n *=87
Clinical setting (*n* = 271), (%)			
Tertiary or university hospital	7.9	62.5	13.6
General hospital	13.2	31.3	86.4
Specialized hospital	9.2	6.3	0
Private practice setting	69.7	0	0

### Discussing sexuality

Table 2 illustrates how often neurologists discuss the quality of the patients’ sexual health. It particularly presents the influence of gender and the patients’ age on bringing up this topic either by the neurologist, the patient, or the partner. The majority of both German and Spanish neurologists report that they address sexuality at least on a regular base with their male patients (61.7% and 78.9%, respectively, with the categories ‘regularly’ and ‘often’ combined). In contrast, only 44.3% of the Dutch respondents stated that they discuss sexuality in the majority of male patients. As for female patients, 68.8% of German and 78.1% of Spanish respondents formulated that they never or seldom talk about possible SD with female PD patients (*p* < 0.001). Similar results were found in the Dutch study. Neurologists in all three countries showed a tendency of discussing sexuality less frequently in elderly patients. In every country, more than 70% of participants replied that they never or seldom address SD in patients aged above 70 years of age (*p* < 0.001). Table 3 shows how frequently sexuality is discussed dependent on the use and efficacy of antiparkinsonian medication and amount of NMS. The usage of a dopamine agonist and the presentation of a large number of other NMS were the most important reasons for neurologists in all three countries for bringing up sexuality during a consultation visit.

**Table 2:** Discussing sexuality with PD patients, total results, and results in subgroups according to gender and age

(%)	Germany				Spain				The Netherlands			
	Never	Seldom	Regularly	Often	Never	Seldom	Regularly	Often	Never	Seldom	Regularly	Often
Male patients^a^	4.4	34.0	45.3	16.4	0	25.0	62.5	12.5	19.3	36.4	22.7	21.6
Female patients^a^	11.3	57.5	23.8	7.5	3.1	75.0	15.6	6.3	38.6	42.0	10.2	9.1
Age (years) (%)												
30–40^a^	6.4	31.2	39.7	22.7	0	29.0	45.2	25.8	0	44.9	47.4	7.7
40–50^a^	4.7	28.0	44.0	23.3	0	21.9	53.1	25.0	0	41.9	50.0	8.1
50–60^a^	3.8	33.3	46.2	16.7	0	21.9	59.4	18.8	2.3	41.4	49.4	6.9
60–70^a^	5.1	46.8	36.1	12.0	0	58.1	35.5	6.5	11.5	44.8	37.9	5.7
> 70^a^	12.1	61.8	20.4	5.7	0	76.7	23.3	0	16.1	55.2	24.1	4.6

^a^N differs, because questions were skipped or forgotten; the categories ‘never’, ‘seldom’, ‘regularly’, and ‘often’ were considered comparable to the categories ‘in less than half of the cases’, ‘in half of the cases’, ‘in more than half of the cases’, and ‘always/almost always’, respectively, used in the Dutch survey

**Table 3:** Discussing sexuality with PD patients in subgroups according to use and efficacy of medication and NMS (*n* = 280)

(%)	Germany	Spain	The Netherlands
Patients not using any antiparkinsonian drugs	23.1	34.4	29.5
Patients using a dopamine agonist^a^	81.9	78.1	77.3
Patients using antiparkinsonian drugs other than a dopamine agonist^a^	44.4	50.0	28.4
Patients with good motor response to medication	25.0	31.3	21.6
Patients with poor motor response to medication	30.0	37.5	22.7
Patients with a lot of non-motor symptoms^a^	52.5	62.5	45.5
Never	3.8	3.1	4.5
Other^b^	26.3	15.6	25.0

^a^Exceeds 100%, because multiple answers were possible

^b^In case of ‘Other’, the majority mentioned “when patients bring it up themselves” (*n* = 25) or “in all patients” (*n* = 18)

Moreover, in all countries, neither patients nor partners frequently expressed sexual problems spontaneously (90.9% and 88.8%, respectively) and often partners were not invited along when SD was discussed (78.6%).

### Barriers

Barriers that neurologists may experience to address sexuality are described in Table 4. Patients not expressing SD spontaneously and a lack of consultation time are the most reported barriers in all three countries, with Spanish participants reporting both of these barriers most frequently (*p* < 0.001 and *p* = 0.001, respectively). The majority of participants do not consider age difference between the patient and neurologist a possible barrier.

**Table 4:** Barriers towards discussing sexuality, sorted from most to least agreed on

(%)	Germany			Spain			The Netherlands		
	Agree	Indecisive	Disagree	Agree	Indecisive	Disagree	Agree	Indecisive	Disagree
Patients do not express sexual problems spontaneously^a^	52.9	32.7	14.4	75.0	12.5	12.5	36.4	21.6	42.0
Insufficient time^a^	32.2	34.9	32.9	71.9	12.5	15.6	37.5	30.7	31.8
High age of the patients^a^	34.0	36.6	29.4	40.6	21.9	37.5	42.0	26.1	31.8
Barriers based on language/culture/religion^a^	30.9	36.2	32.9	34.4	12.5	53.1	24.1	25.3	50.6
Patient is not ready to discuss sexuality^a^	20.1	55.8	24.0	21.9	40.6	37.5	10.2	34.1	55.7
Patient is too ill to discuss sexuality^a^	26.0	44.0	30.0	12.5	28.1	59.4	18.2	17.0	64.8
Insufficient training/knowledge^a^	6.7	20.1	73.2	15.6	34.4	50.0	18.4	50.6	31.0
Patient is of the opposite sex^a^	11.2	19.1	69.7	15.6	12.5	71.9	5.7	10.2	84.1
Age difference between yourself and the patient^a^	8.4	9.1	82.5	18.8	3.1	78.1	6.8	9.1	84.1
I feel uncomfortable to talk about sexuality^a^	3.3	25.0	71.7	12.9	29.0	58.1	14.8	34.1	51.1
Someone else is accountable for discussing sexuality^a^	3.3	8.7	71.7	12.5	25.0	58.1	5.7	18.4	51.1

^a^N differs, because questions were skipped or forgotten

### Screening

Screening for SD in PD was rated as ‘important’ or ‘very important’ by 68.3% of German participants and 96.9% of Spanish respondents. Nevertheless, the majority reported to ‘(almost) never’ or ‘in less than half of the cases’ use any questionnaire or tool to assess NMS, including SD (86.9% in German participants and 75% in Spanish participants). These results are similar to the Dutch study.

### Training and knowledge on the topic

The topic of SD is implemented in the training program of neurology residents in Germany according to 53.8% of the German respondents, while 90.6% of the Spanish participants replied this is not the case in their country (Supplementary Table 5). Both in Germany and Spain, the majority of the participants considered themselves competent (87.3% and 62.5%, respectively) to discuss the topic of sexuality with their patients (supplementary Table 6). On the other hand, most of them (70.4% and 96.9%, respectively) welcomed additional training to extend their knowledge on SD in PD, comparable to their Dutch colleagues (supplementary Table 7). Higher levels of knowledge lead to addressing sexuality more frequently (*p* = 0.004). Participants also reported a need for information materials on the subject (Supplementary Table 15), such as brochures to hand out to patients (*n* = 149, 78.0%).

### Responsibility

**Fig. 1:**
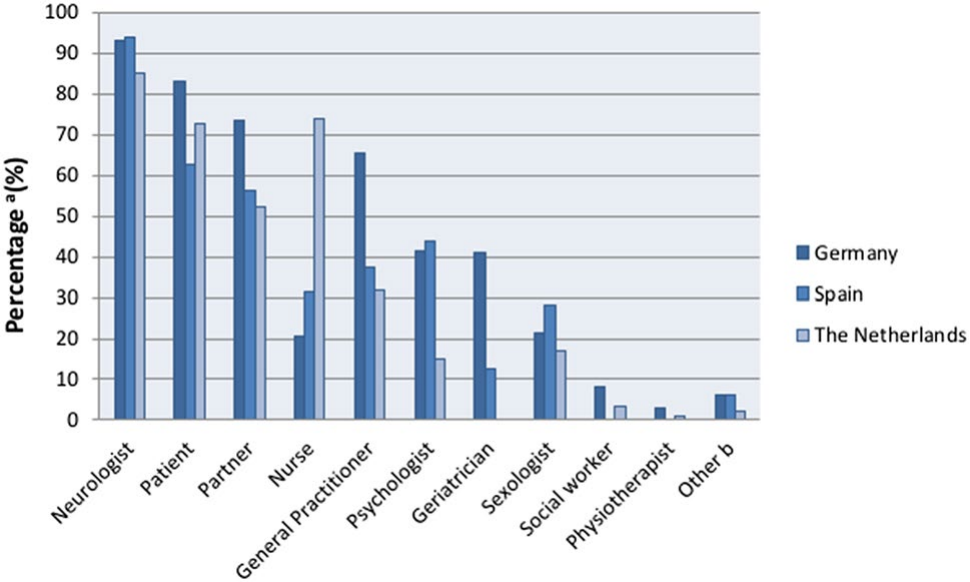
Responsibility for discussing sexuality. **a** Exceeds 100%, because multiple answers were possible. **b** ‘Other’ includes ‘Urologist’ (*n* = 7) and ‘Gynaecologist’ (*n* = 3)

In all three countries, more than 85% of the respondents considered the treating neurologist responsible for addressing sexual health during a patient visit (Fig. 1). The majority of the respondents also stated that the patient and partner are responsible for bringing up problems in the patients’ sexual functioning. Contrary to the Dutch participants, only a minority of German and Spanish neurologists (20.8% and 31.3%, respectively) feel their Parkinson’s nurse should address sexuality during a patient consultation.

According to German and Spanish respondents, the vast majority of their centres (96.2% in Germany and 81.3% in Spain) do not have a protocol making it obligatory for physicians to discuss sexuality (Supplementary Table 11). There are also no clear agreements within many departments which care provider is responsible for addressing this topic (*n* = 151, 82.3% of German participants and 70.0% of Spanish participants; supplementary Table 12).

Remaining survey results are presented in Online Resource 2.

## Discussion

In a previous study, we demonstrated that while most Dutch neurologists consider sexuality an important topic to discuss with their PD patients, the majority of them fail to address sexuality related issues with their patients on a routine basis (van Hees et al. 2017). We questioned whether cultural psychological factors underlie this lack of discussing SD and performed a similar survey in Germany and Spain. Interestingly, we found that sexuality was much less discussed with female patients. This trend was also seen in the Dutch survey, but was much more striking in the other two countries, especially Spain. In fact, both in Germany and Spain, over half of the neurologists discuss SD regularly or often with their male patients, while for female patients, it is the other way around. In a search for an explanation for this remarkable difference, we analysed the background of the respondents and noticed that the vast majority of German participants work in a private practice, while most of the Spanish participants work in a university or tertiary hospital. Hence, the setting of the neurologists’ practice probably does not explain this difference. Interestingly, most of the participants in Germany were male, while in Spain, the majority was female. As approximately 70% of the Spanish and German respondents do not consider the opposite sex of patients a barrier towards discussing SD, we conclude that it is unlikely that this will explain why German neurologists discuss sexuality less frequently with female patients. However, we cannot exclude that the gender of Spanish female neurologists influences their decision to address sexuality less often with female patients, as we did not ask the participants whether they consider patients having the same gender a barrier. We also cannot rule out the influence of cultural differences between both countries. On the other hand, the undervaluation of female SD is seen across several other diseases and countries (Lew-Starowicz and Rola 2013; Arango-Lasprilla et al. 2017; Wasner et al. 2004).

One of our key findings was that the largest barrier neurologists experience towards discussing sexuality, is patients not raising the topic themselves, which was particularly found for Spanish respondents. We consider this problematic as the majority of our participants reported that patients almost never bring up sexual problems spontaneously, and patients’ partners never or rarely do so either. Although the majority of respondents want patients to initiate the discussion of sexual problems themselves, almost all participants report that the neurologist should be responsible for doing so, together with the patient and their partner. This preference for a shared responsibility by patient, partner, and physician was found in all three countries. However, differences were seen as well, with Dutch physicians describing a clear role for nurses, while German physicians were more likely to include geriatricians or psychologists in discussing SD. Different ways’ healthcare is organized in the three countries may explain these variations. While in The Netherlands, most PD patients are treated by neurologists, PD patients in Germany and Spain may be seen by neurologists, family physicians, geriatricians, and other doctors. In The Netherlands, PD nurse specialists already play an important role in the patient’s treatment, while in Spain, there are only few PD nurses, and in Germany, PD nurses are increasingly employed in movement disorder units of university hospitals or large community hospitals, but only rarely in private practices. These (graduated) nurses have attended training courses, which are specifically designed to expand their knowledge and skills on care and treatment of PD patients (Lennaerts et al. 2017). Usually, patients consider nurses to be more accessible as they tend to have closer contact with the patient and also have longer consultation time (Frundt et al. 2018). This may make them a good candidate to discuss sexuality, as another frequently reported barrier by physicians was insufficient time during consultation. Especially, a large number of Spanish participants considered this a problem, with the majority of them working in an academic hospital. As most German respondents work in a private clinic, one may question whether the organization of medical service may be of influence on the decision whether or not to discuss sexual dysfunction with PD patients.

A possible solution for making it easier and more time efficient for physicians and patients to talk about sexuality is to implement the use of a tool to track PD symptoms, such as the Parkinson Monitor which is often applied in Dutch practices. We also recommend composing local guidelines that define who is responsible for addressing SD and how patients can be referred to caregivers specializing in sexual health care. A strategy which may be of added value is making patients check off which complaints they experience on a list of possible PD symptoms before they visit their physician and select a top three of most pressing problems to discuss with their physician. Remaining symptoms will be discussed during a following consultation visit with the PD nurse specialist. As SD is included in the list of symptoms, patients will likely be less hesitant to report on this topic, and as such, SD can be mentioned without patients having to actually initiate the conversation themselves. Moreover, this would provide a solution to the most frequently reported barrier of patients not expressing sexual complaints spontaneously.

The lack of discussing SD could be twofold, with patient and physician both seeming to be reluctant to raise the topic and hoping and expecting the other party to initiate the conversation. Both components of this problem could be the result of a lack of knowledge on the caregiver’s side. This may be improved by implementing the topic of SD within training programs as obligatory material, expand the number of courses on sexuality and compose local guidelines on PD related SD. Although about half of our respondents reported that they did not experience insufficient training or knowledge as a barrier in discussing sexuality and rated their level of knowledge on SD as ‘some’, the majority feels that it is necessary to increase their knowledge on discussing sexual problems. German and Spanish physicians declared the largest need for increasing their knowledge. In addition, a large majority of Spanish participants would like to receive more training on the subject of sexuality, which is interesting, as most of them worked in a university hospital. Furthermore, when asked what may improve discussing sexuality, most participants answered that they would like brochures on SD to hand over to their patients. It is demonstrated that when patients are provided with information and education on SD, they will feel more motivated and confident to discuss the subject (Mellor et al. 2013). The same has been found in physicians, i.e., when knowledge and training on SD increased, physicians were more likely to address sexual functioning and apply interventional procedures (Dyer and das Nair 2013; Miller and Byers 2012). This is in line with our results, as physicians did not just report a need for an increase in their own knowledge and education on sexuality, but also welcomed more materials to inform patients about SD related issues that may occur. We consider professional and patient networks as ideal platforms to provide these materials.

All in all, there is a stark gap between the importance that physicians assign to sexual problems in PD and how often they discuss these issues in their daily clinical practice. We demonstrated a need to improve sexual health care for PD patients across all countries studied, which likely means that the disparity between importance and discussing sexual health exists in other European countries as well. We propose to implement a tool to track PD symptoms that gives both neurologists and PD nurses the opportunity and responsibility to discuss sexuality and increase their knowledge on this topic.

A survey-based study such as this one has a couple of limitations. First of all, because this study was based on self-report, there might be an overestimation of answers physicians consider socially desirable. In an attempt to reduce this bias, the surveys were anonymous. Moreover, due to the nature of the study, there might be a recall bias and non-response bias as well. Regarding the latter, no comparisons could be made between respondents and non-respondents, as demographics of non-respondents were unknown. A lack of interest in the topic could be the reason why physicians refused to fill in the survey. As such, one may assume that in daily practice, sexual dysfunction is discussed even less than we found in our study.

Similar to the previous Dutch study, a non-validated questionnaire was used, which may be regarded as another limitation (van Hees et al. 2017). Finally, a comparison of the survey results of the three countries may be confounded due to variances in translation of the questionnaires.

## Conclusion

Across Germany, Spain, and The Netherlands, neurologists do not regularly discuss sexuality with PD patients, especially female patients. Our findings suggest that patients not expressing sexual problems spontaneously, together with insufficient consultation time, are the most important factors in not addressing sexual matters. Applying a tool that patients can use to track and rate their PD symptoms and sharing the responsibility of discussing sexuality between physicians and nurses may improve sexual care for PD patients.

In each of the three countries, the majority of the physicians reported the need to increase their own and patients’ knowledge on the topic. Professional and patient networks may be helpful in providing education materials.

## Electronic supplementary material

Below is the link to the electronic supplementary material.

Questionnaire (English version) (PDF 189 kb)

Supplementary data (PDF 250 kb)
