# Supplementary material for: Discussing sexuality with Parkinson’s disease patients: a multinational survey among neurologists
Source: J Neural Transm (Vienna). 2019 Aug 8;126(10):1273–80. doi: 10.1007/s00702-019-02053-5 (PMC6765045; doi:10.1007/s00702-019-02053-5)
Supplement: Supplementary file 1 — Questionnaire (English version) (PDF 189 kb) [file 702_2019_2053_MOESM1_ESM.pdf]

Supplementary data to 'Discussing sexuality with Parkinson's disease patients: a multinational survey among neurologists'

Journal of Neural Transmission

F.B.B. de Rooy, MD[a]; C. Buhmann, MD, PhD[b]; B. Schönwald, MSc[b]; P. Martinez-Martin, MD, PhD[c]; C. Rodriguez-Blazquez, PhD[c], H. Putter, PhD[d]; H.W. Elzevier, MD, PhD[a]; A.A. van der Plas MD, PhD[e]

Corresponding author: Frédérique B.B. de Rooy, Department of Urology, Leiden University Medical Center, Albinusdreef 2, PO Box 9600, Leiden 2300 RC, The Netherlands. Tel: (0031) 071-5265255; Fax: (0031) 071-5248135; E-mail: F.B.B.de_Rooy@lumc.nl

In case you do not want to participate, please indicate the reason:
☐ No time
☐ Not interested
☐ Not enough experience
☐ Research is not important in this area
☐ No enhancement possible in this area
☐ Retired
☐ Research is not important in this area
☐ Other: ………………………………………

Questionnaire

Please read the questionnaire carefully. Choose the most suitable answer at each question. Please do not skip any questions. In case of mistakes, please cross out the wrong answer and select the right box. Thank you in advance for your effort.

Demographics

1. What is your gender?
o Male
o Female

2. What is your age? _________ Years

3. What is your current position at work?
o Neurologist
o Geriatrician
o Other, namely: ____

4. Type of clinic/practice?
o Tertiary or university hospital
o General hospital
o Specialized hospital (for instance a cancer center)

5. Time of practice in neurology?
o < 1 year
o 1-2 years
o 3-5 years
o 6-10 years
o 11-15 years
o > 15 years

The following questions concern sexuality in Parkinson's disease

6. In which patients suffering from Parkinson's disease do you discuss sexuality? (Multiple answers possible)
☐ Patients not using any antiparkinsonian drugs
☐ Patients using a dopamine agonist
☐ Patients using antiparkinsonian drugs other than a dopamine agonist
☐ Patients with good motor response to medication
☐ Patients with poor motor response to medication
☐ Patients with a lot of non-motor symptoms
☐ Never
☐ Other: ____

7. How often do you discuss sexual health with patients suffering from Parkinson's disease during the first consultation?
o Never/almost never
o In less than half of the cases
o In half of the cases
o In more than half of the cases
o Almost always/always

8. How often do you discuss sexual health with patients suffering from Parkinson's disease during follow-up?
o Never/almost never
o In less than half of the cases
o In half of the cases
o In more than half of the cases
o Almost always/always

9. How often do you discuss altered sexuality (hypersexuality) as adverse drug reaction on dopamine replacement therapy (especially dopamine agonists)?
- Never/almost never
- In less than half of the cases
- In half of the cases
- In more than half of the cases
- Almost always/always

10. How often do you discuss sexual health with male and female patients suffering from Parkinson’s disease?

Gender	Never	Seldom	Regularly	Often
Male	□	□	□	□
Female	□	□	□	□

11. How often do you discuss sexual health with patients suffering from Parkinson’s disease in the age groups listed below?

Age groups	Never	Seldom	Regularly	Often
30-40 years	□	□	□	□
40-50 years	□	□	□	□
50-60 years	□	□	□	□
60-70 years	□	□	□	□
> 70 years	□	□	□	□

12. To what extent do you use the ‘Parkinson Well-being Map’ or other questionnaires (for example the non-motor symptom questionnaire) to assess non-motor manifestations, including sexual dysfunctions, in patients suffering from Parkinson’s disease?
- Never/almost never
- In less than half of the cases
- In half of the cases
- In more than half of the cases
- Almost always/always

13. How often do patients express sexual problems spontaneously?
- Never/almost never
- In less than half of the cases
- In half of the cases
- In more than half of the cases
- Almost always/always

14. How often do patients’ partners express sexual problems spontaneously?
- Never/almost never
- In less than half of the cases
- In half of the cases
- In more than half of the cases
- Almost always/always

15. How often do you invite the partner of the patient when you discuss sexuality?
- Never/almost never
- In less than half of the cases
- In half of the cases
- In more than half of the cases
- Almost always/always

16. Possible barriers towards discussing sexuality are listed below. To what extent are the barriers applicable to you? Please select only one answer for each barrier.

	Totally disagree	Disagree	Slightly disagree/ slightly agree	Agree	Totally agree
I feel uncomfortable to talk about sexuality	□	□	□	□	□
Insufficient time	□	□	□	□	□
Insufficient training/knowledge	□	□	□	□	□
Someone else is accountable for discussing sexuality	□	□	□	□	□
Patient is not ready to discuss sexuality	□	□	□	□	□
Patient is too ill to discuss sexuality	□	□	□	□	□
Patients do not express sexual problems spontaneously	□	□	□	□	□
Barriers based on language/culture/religion	□	□	□	□	□
Patient is of the opposite sex	□	□	□	□	□
High age of the patients	□	□	□	□	□
Age difference between yourself and the patient	□	□	□	□	□

17. Is it, according to a protocol at your department/centre, obligatory to discuss sexuality?
- o Yes
- o No
- o I don't know

18. Are there clear agreements made within your department regarding which care provider is responsible for discussing sexuality?
- o Yes, namely: ____
- o No
- o Unaware

19. Is it possible to refer patients experiencing sexual problems to other care providers in your clinic?
- o Yes, to: ____
- o No
- o Unknown

20. What percentage of your patients suffering from Parkinson's disease did you refer to another care provider for counselling of their sexual problems over the last year? ______________%

The following questions concern knowledge on sexuality in Parkinson's disease

21. Is the subject 'sexual dysfunction in Parkinson's disease' implemented in the training program of neurology/geriatric residents?
- o Yes
- o No

22. How do you rate your own knowledge on sexual dysfunctions and the treatment of it?
- o No knowledge at all
- o Insufficient knowledge
- o Some knowledge
- o Sufficient knowledge
- o A lot of knowledge

23. Do you feel competent to discuss sexuality to patients with Parkinson's disease?
- o Yes
- o No

24. Are you in need of extending your knowledge on discussing sexuality?
- o Yes
- o No

25. Are you aware of the existence of the National Clinical Guideline for Diagnosis and Management of Parkinson's disease of The National Collaborating Centre for Chronic Conditions?
o Yes
o No

26. Are you aware of the chapter on sexual dysfunctions in this guideline?
o Yes
o No

27. How often do you apply this chapter on sexual dysfunctions in daily practice?
o Never/almost never
o In less than half of the cases
o In half of the cases
o In more than half of the cases
o Almost always/always
o I am not aware of the existence of this chapter about sexual dysfunctions

28. What facilities might enhance the discussion on sexuality? (Multiple answers possible)
☐ Brochures on sexuality to hand over to patients
☐ Online applications or paper questionnaires
☐ Training on how to discuss sexuality
☐ Posters in the waiting room
☐ A list of care providers to whom patients with sexual problems can be referred to
☐ Implementation of an education course on sexual dysfunction in Parkinson's disease in the training program of neurology/geriatric residents
☐ Other, namely:____

The following questions concern your opinion about discussing sexuality in Parkinson's disease

29. Who is, according to you, responsible for discussing sexuality? (Multiple answers possible)
☐ Patient
☐ Partner of the patient
☐ Neurologist
☐ Geriatrician
☐ General practitioner
☐ Social worker
☐ Nurse
☐ Psychologist
☐ Physiotherapist
☐ Sexologist
☐ Other, namely: ____

30. How important is it to pay attention to sexual dysfunction in Parkinson's disease?
o Unimportant
o Slightly important
o Important
o Very important
o Indecisive

Thank you very much for completing the questionnaire.
