# Supplementary material for: Discussing sexuality with Parkinson’s disease patients: a multinational survey among neurologists
Source: J Neural Transm (Vienna). 2019 Aug 8;126(10):1273–80. doi: 10.1007/s00702-019-02053-5 (PMC6765045; doi:10.1007/s00702-019-02053-5)
Supplement: Supplementary file 2 — Supplementary data (PDF 250 kb) [file 702_2019_2053_MOESM2_ESM.pdf]

Supplementary data to 'Discussing sexuality with Parkinson's disease patients: a multinational survey among neurologists'

Journal of Neural Transmission

F.B.B. de Rooy, MD[a]; C. Buhmann, MD, PhD[b]; B. Schönwald, MSc[b]; P. Martinez-Martin, MD, PhD[c]; C. Rodriguez-Blazquez, PhD[c], H. Putter, PhD[d]; H.W. Elzevier, MD, PhD[a]; A.A. van der Plas MD, PhD[e]

Corresponding author: Frédérique B.B. de Rooy, Department of Urology, Leiden University Medical Center, Albinusdreef 2, PO Box 9600, Leiden 2300 RC, The Netherlands. Tel: (0031) 071-5265255; Fax: (0031) 071-5248135; E-mail: F.B.B.de_Rooy@lumc.nl

Supplementary table 1 Frequency of patients and partners expressing sexual problems spontaneously (n=280 and n=192 respectively)

	**Germany**		**Spain**		**The Netherlands**	
	Patient	Partner	Patient	Partner	Patient	Partner
Never/almost never (%)	42.5	54.4	34.4	28.1	47.7	n/a [a]
In less than half of the cases (%)	55.0	41.9	50.0	53.1	43.2	n/a [a]
In half of the cases (%)	4.0	3.1	6.3	12.5	8.0	n/a [a]
In more than half of the cases (%)	0	0.6	6.3	3.1	1.1	n/a [a]
Almost always/always (%)	0	0	3.1	3.1	0	n/a [a]

[a] This question was not included in the Dutch survey

Supplementary table 2 Frequency of inviting the patients' partner along when discussing sexuality (n=192)

	**Germany**	**Spain**	**The Netherlands**
Never/almost never (%)	51.9	12.5	n/a [a]
In less than half of the cases (%)	31.3	43.8	n/a [a]
In half of the cases (%)	7.5	6.3	n/a [a]
In more than half of the cases (%)	6.3	25.0	n/a [a]
Almost always/always (%)	3.1	12.5	n/a [a]

[a] This question was not included in the Dutch survey

Supplementary table 3 Level of importance assigned to sexual dysfunction in PD (n=278)

	**Germany**	**Spain**	**The Netherlands**
Unimportant (%)	4.4	0	1.1
Slightly important (%)	23.4	3.1	37.5
Important (%)	54.4	65.6	47.7
Very important (%)	13.9	31.3	11.4
Indecisive (%)	3.8	0	2.3

Supplementary table 4 Extent to which questionnaires are used to assess non-motor manifestations, including sexual dysfunction, in PD patients (n=280)

	**Germany**	**Spain**	**The Netherlands**
Never/almost never (%)	73.8	37.5	47.7
In less than half of the cases (%)	13.1	37.5	26.1
In half of the cases (%)	4.4	9.4	10.2
In more than half of the cases (%)	4.4	12.5	12.5
Almost always/always (%)	4.4	3.1	3.4

Supplementary table 5 Is sexuality included in training programs of neurology residents? (n=175)

	Germany	Spain	The Netherlands
Yes (%)	53.8	9.4	n/a [a]
No (%)	46.2	90.6	n/a [a]

[a] This question was not included in the Dutch survey

Supplementary table 6 Do neurologists feel competent to discuss sexuality with their PD patients? (n=190)

	Germany	Spain	The Netherlands
Yes (%)	87.3	62.5	n/a [a]
No (%)	12.7	37.5	n/a [a]

[a] This question was not included in the Dutch survey

Supplementary table 7 Are neurologists in need of extending their knowledge on discussing sexuality? (n=279)

	**Germany**	**Spain**	**The Netherlands**
Yes (%)	70.4	96.9	56.8
No (%)	29.6	3.1	43.2

Supplementary table 8 Level of knowledge on sexual dysfunction and its treatment (n=278)

	**Germany**	**Spain**	**The Netherlands**
No knowledge at all (%)	0.6	0	2.3
Insufficient knowledge (%)	12.0	31.3	22.7
Some knowledge (%)	43.0	43.8	63.6
Sufficient knowledge (%)	39.9	25.0	10.2
A lot of knowledge (%)	4.4	0	1.1

Supplementary table 9 Are neurologists aware of the existence of the National Clinical Guideline for Diagnosis and Management of Parkinson's Disease (NCGDMPD) and the chapter on sexual dysfunction in it?

	**Germany**		**Spain**		**The Netherlands**	
	Yes (%)	No (%)	Yes (%)	No (%)	Yes (%)	No (%)
Aware of the NCGDMPD guideline [a]	15.1	84.9	87.5	12.5	n/a [b]	n/a [b]
Aware of the chapter on SD [a]	15.8	84.2	51.6	48.4	n/a [b]	n/a [b]

[a] N differs because questions were skipped or forgotten

[b] This questions was not included in the Dutch survey

Supplementary table 10 Frequency of using the NCGDMPD guideline's chapter on SD (n=183)

	**Germany**	**Spain**	**The Netherlands**
Never/almost never (%)	68.2	34.4	n/a [a]
In less than half of the cases (%)	11.9	12.5	n/a [a]
In half of the cases (%)	2.6	0	n/a [a]
In more than half of the cases (%)	0.7	12.5	n/a [a]
Almost always/always (%)	0.7	12.5	n/a [a]
Not aware of the existence of this chapter (%)	15.9	28.1	n/a [a]

[a] This question was not included in the Dutch survey

Supplementary table 11 Are there protocols at participants' department/centre making it obligatory to discuss sexuality? (n=189)

	**Germany**	**Spain**	**The Netherlands**
Yes (%)	3.2	3.1	n/a [a]
No (%)	96.2	81.3	n/a [a]
Unaware (%)	0.6	15.6	n/a [a]

[a] This question was not included in the Dutch survey

Supplementary table 12 Are there clear agreements made within participants' department regarding which care provider is responsible for discussing sexuality? (n=188)

	**Germany**	**Spain**	**The Netherlands**
Yes [a] (%)	16.5	6.7	n/a [b]
No (%)	82.3	70.0	n/a [b]
Unaware (%)	1.3	23.3	n/a [b]

[a] In case of 'Yes', most neurologists mentioned 'the treating physician' (n=23)

[b] This question was not included in the Dutch survey

Supplementary table 13 Is referral of patients experiencing sexual problems to other care providers in your clinic possible? (n=280)

	**Germany**	**Spain**	**The Netherlands**
Yes [a] (%)	29.6	71.9	77.3
No (%)	66.4	15.6	20.5
Unaware (%)	3.9	2.3	2.3

[a] In case of 'Yes', neurologists mentioned 'urologist' (n=67), 'gynaecologist' (n=15), 'sexologist' (n=15) and 'psychologist' (n=13)

[b] This question was not included in the Dutch survey

Supplementary table 14 Percentage of PD patients referred to another care provider for sexual complaints over the last year (n=249)

	**Germany**	**Spain**	**The Netherlands**
Mean percentage (standard deviation)	6.5 (14.0)	10.2 (8.6)	6.2 (9.6)

Supplementary table 15 Facilities that may improve the discussion of sexuality (n=191)

	**Germany**	**Spain**	**The Netherlands**
Brochures on sexuality to hand over to patients [a] (%)	78.6	75.0	n/a [c]
Online applications or paper questionnaires [a] (%)	32.7	25.0	n/a [c]
Training on how to discuss sexuality [a] (%)	32.7	71.9	n/a [c]
Posters in the waiting room [a] (%)	11.9	12.5	n/a [c]
A list of care providers to whom patients with SD can be referred [a] (%)	49.1	50.0	n/a [c]
Implementation of an education course on SD in training program of neurology residents [a] (%)	47.2	59.4	n/a [c]
Other [a, b] (%)	10.1	3.1	n/a [c]

[a] Exceeds 100% because multiple answers were possible

[b] In case of 'Other', neurologists mentioned 'more consultation time' (n=5)

[c] This question was not included in the Dutch survey
